# Airborne Radar Anti-Jamming Waveform Design Based on Deep Reinforcement Learning

**DOI:** 10.3390/s22228689

**Published:** 2022-11-10

**Authors:** Zexin Zheng, Wei Li, Kun Zou

**Affiliations:** Information and Navigation College, Air Force Engineering University, Xi’an 710077, China

**Keywords:** radar anti-jamming, waveform design, deep reinforcement learning

## Abstract

Airborne radars are susceptible to a large number of clutter, noise and variable jamming signals in the real environment, especially when faced with active main lobe jamming, as the waveform shortcut technology in the traditional regime can no longer meet the actual battlefield radar anti-jamming requirements. Therefore, it is necessary to study anti-main-lobe jamming techniques for airborne radars in complex environments to improve their battlefield survivability. In this paper, we propose an airborne radar waveform design method based on a deep reinforcement learning (DRL) algorithm under clutter and jamming conditions, after previous research on reinforcement-learning (RL)-based airborne radar anti-jamming waveform design methods that have improved the anti-jamming performance of airborne radars. The method uses a Markov decision process (MDP) to describe the complex operating environment of airborne radars, calculates the value of the radar anti-jamming waveform strategy under various jamming states using deep neural networks and designs the optimal anti-jamming waveform strategy for airborne radars based on the duelling double deep Q network (D3QN) algorithm. In addition, the method uses an iterative transformation method (ITM) to generate the time domain signals of the optimal waveform strategy. Simulation results show that the airborne radar waveform designed based on the deep reinforcement learning algorithm proposed in this paper improves the signal-to-jamming plus noise ratio (SJNR) by 2.08 dB and 3.03 dB, and target detection probability by 26.79% and 44.25%, respectively, compared with the waveform designed based on the reinforcement learning algorithm and the conventional linear frequency modulation (LFM) signal at a radar transmit power of 5 W. The airborne radar waveform design method proposed in this paper helps airborne radars to enhance anti-jamming performance in complex environments while further improving target detection performance.

## 1. Introduction

### 1.1. Background and Motivation

The rapid development of airborne radar has eliminated the effects of the ground environment and the curvature of the earth on surface-to-air radar. Airborne radar has expanded the detection distance of various low-altitude and ultra-low-altitude targets on the ground, in the air and at sea, and has become one of the key factors of battlefield victory [1]. Airborne radar has the advantages of small blind spots, strong manoeuvrability and high resolution. It can detect long-range targets in all directions and around the clock, control and guide weapons and undertake tasks such as air alert, reconnaissance and flight safety. It plays an important role in land–air defence.

With the continuous development of communication technology and electronic warfare technology, there are many types of electronic equipment on the modern battlefield, and there are situations where the frequency bands used by electronic equipment overlap and interfere with each other. With the rapid development of manufacturing technology and the continuous upgrading of enemy jamming equipment, airborne radar must not only overcome the impact of terrain clutter caused by the complex geographical environment, but also the diversified, dexterous and intelligent jamming of aircraft and missiles that are rushing to attack. These include passive jamming techniques such as foil jamming and towed jamming; active jamming techniques such as deception jamming; and suppression jamming, for which there are a variety of implementation methods, such as long-range support, follow-on and self-defence jamming. Strong clutter and jamming signals in complex environments can reduce the probability of radar target detection by drowning out target echo information, as well as blurring the target signal and reducing the probability of target identification, or forming false targets and increasing the probability of false alarms. These jamming techniques all pose a challenge to airborne radars performing tasks such as search, identification and guidance [2].

Traditional anti-main-lobe jamming techniques include passive anti-jamming methods such as null-domain zeroing, frequency agility and phase encode technology. In particular, the airspace anti-jamming method [3] uses an antenna array to zero in a particular direction of the antenna direction diagram, thereby suppressing side lobe jamming from that direction, but when the target performs self-defence jamming, the jamming and target echoes come from the same angle, i.e., direction, and the radar is unable to detect the target while suppressing the main lobe self-defence jamming by zeroing at the same time. When an airborne radar is subjected to main lobe jamming, the detection probability can be improved by changing the detection method and detection threshold to reduce the probability of false alarms on the radar when the jamming signal has a specific distribution and the jamming power is low. However, this method has difficulty coping with high-power jamming and adapting to jamming from changing parameters such as signal distribution and energy spectra. It can be seen that anti-jamming means starting from airspace zeroing and detection techniques are hardly effective in suppressing main lobe jamming. Airborne radars using these means have low flexibility in transmitted waveforms, fixed received signal processing and a very limited anti-jamming capability, which make it difficult to meet the requirements of a non-uniform, non-smooth radar operating environment [4] and pose a huge threat to airborne radars performing target detection, tracking and guidance tasks. To ensure the good performance of airborne radars in complex electromagnetic environments, it is necessary to research radar anti-main-lobe jamming.

Designing and transmitting waveforms based on cognitive radar concepts [5] that match target characteristics and jamming spectral features can overcome the limitations of traditional methods, such as improving performance from the receiver side only or randomly shortcutting waveform parameters at the transmitter side, thereby radically improving radar detection performance in main lobe jamming conditions. Since then, waveform design based on cognitive radar thinking has gradually become a hot research topic in the field of waveform design.

This paper aims to design an optimal anti-jamming waveform in a complex and changing electromagnetic environment, based on the electromagnetic environment information acquired by the radar in advance and the mission requirements of the radar, and to lay the theoretical foundation for promoting the development of active anti-jamming technology for radars.

### 1.2. Related Works

Active radar jamming techniques seriously degrade the target detection, tracking and identification performance of radars and are the biggest threat to radar survival today. Therefore, it is necessary to study radar anti-active jamming techniques. With the improvement in hardware performance and in-depth research on signal processing technology, waveform design is favoured by radar researchers at home and abroad as one of the most effective ways to improve the overall performance of the radar.

Waveform shortcutting is valued for its anti-jamming properties in the face of main lobe jamming [6]. The main ways of dealing with jamming from a waveform perspective are currently frequency shortcuts and phase-coded signal shortcuts. The literature [7] proposed a dense dummy target jamming suppression algorithm based on the joint waveform entropy of shortcut frequency change and provided an outlook on the development trend of frequency shortcut radar waveform countermeasure technology [8]. The literature [9] also proposed an anti-main-lobe jamming method by sensing the jamming environment to achieve low intercept waveform scheduling for radar, which effectively improved the target tracking performance in multiple main lobe jamming environments. In the literature [10], a sparse step linear FM waveform optimization algorithm with frequency and pulse repetition frequency agility was proposed to study the distance and Doppler 2D autocorrelation functions of the shortcut waveform, paving the way for finding a frequency and PRF optimization strategy to suppress the distance and Doppler partials. However, the frequency shortcutting method mostly shortcuts the transmitting frequency in inter- or intra-vehicle according to the hopping pattern [11], and processing the hopping signal within the coherent processing time generates side lobes, resulting in spikes in the Doppler spectrum, and thus increasing the probability of false alarms and reducing the target detection performance of the radar [12]. At the same time, the airborne radar hopping pattern has been preset before working and cannot be changed in real time according to the target and jamming environment, resulting in sometimes jumping into the jamming signal coverage band, which in turn increases the possibility of jamming.

Phase-coded signals are also a common form of signal used for shortcuts [13]. Through rational design, the phase-coded signal can have a high autocorrelation function main-to-side-lobe ratio and also a high mutual quadrature performance, which provides good anti-jamming performance when the jammer cannot keep up with the transmitter changes in time or cannot forward the jamming signal in real time. For co-channel jamming, the literature [14] proposed a phase-coded anti-jamming method in conjunction with the constant false alarm technique. The literature [15] proposed a phase-coded signal system that effectively suppressed the main lobe jamming and improved the target detection probability. In the literature [16], an anti-retransmission jamming technique was proposed using phase- and frequency-coded waveform techniques. To generate effective agile waveforms, an improved Logistic-Map chaotic sequence generation and optimization algorithm was proposed in the literature, using phase and frequency coding techniques to obtain an orthogonal polyphase code set, which effectively suppresses retransmission jamming. To combat repeater jamming, the literature [17] proposes an adaptive transmitting scheme based on phase-coded signals by sensing jamming parameters and using genetic algorithms to optimize the waveform. To address the problem of extended target detection in cluttered and noisy environments, a phase-modulated waveform design method based on a maximum signal-to-noise ratio criterion is proposed in the literature [18]. However, there are significant difficulties in generating a sufficient number of coded signals that are orthogonal to each other. As the number of coded signals increases, the mutual orthogonality of the signals in the set decreases, which can lead to a certain level of jamming performance when forward jamming is based on the previous pulse or pulses.

The above research shows that radar anti-jamming technology has made great progress. However, the existing anti-jamming waveforms are not designed to change in real time according to the environment. The waveforms have limited variation and the echo signal processing is fixed, making it difficult to meet the time-varying, non-smooth and non-uniform operating environment. When airborne radars are confronted with new types of jamming, the practical anti-jamming performance of traditional anti-jamming techniques is limited [4], so there is an urgent need to investigate new waveform design methods to improve radar countermeasures.

Professor Simon Haykin has developed a cognitive radar concept [5] that draws on biological bionic knowledge to design and transmit waveforms that match the characteristics of the target and the jamming spectrum by sensing information about the environment and the target. The cognitive radar concept establishes closed-loop adaptive processing of radar from reception to transmission, overcoming the limitations of conventional radars that can only perform performance enhancements from the receiver side or randomly change transmission parameters at the transmitter side, radically improving airborne radar detection performance under clutter and jamming conditions.

Cognitive radar waveform design, as one of the main ways of active radar anti-jamming at present, can better design radar anti-jamming waveforms adapted to the current environment based on the electromagnetic spectrum characteristics of the signal and the characteristics of the detected target, to improve the detection and identification performance of the radar. Research on radar anti-jamming based on cognitive radar waveform design has yielded fruitful results. To address the extended clutter suppression problem, the literature [19] investigated radar waveform design using inter-pulse phase-encoded signals. For intermittently sampled forwarded jamming, the literature [20] demonstrates the design of constant-mode radar waveforms by reducing the side lobe level. In the context of inaccurate a priori information on both random target impulse response and signal-related clutter, the literature [21] proposed a robust algorithm for the joint design of cognitive radar low peak-to-average ratio transmit waveforms and receiver filters. The literature [22] introduces the basic framework of cognitive radar waveform optimization and systematically compares the main research contents and research progress of waveform optimization for detection, tracking, imaging and classification tasks as well as anti-jamming cognitive waveform optimization, providing a comparative vertical and horizontal perspective for the research of waveform optimization techniques for single tasks and joint multi-tasks. For the problem of adaptive waveform design for extended target recognition in cognitive radar networks, a minimum correlation algorithm is proposed in the literature [23] to adaptively design the transmit waveform for each radar in an amplitude fluctuation situation. However, waveform design based on cognitive radar thinking still requires the establishment of an optimized objective function. In a world where radars are faced with multi-functional and multi-mission requirements, the comprehensive enhancement of radar performance has become an issue that has to be addressed. However, most of the existing waveform design methods use fixed guidelines, which are difficult to adapt to complex electromagnetic environments. Deep reinforcement learning, with its strong ability to interact with the environment, can automate radar waveform design by sensing the input environmental information to design new radar-transmitted waveforms in real time.

Reinforcement learning can be found in many areas of artificial intelligence where decision making and control are required, such as in games and gaming. Reinforcement learning has the ability to interact with the environment in real time and to make optimal decisions by continuously learning information about the environment, and has been successfully applied in many areas, such as Go, gaming and medical diagnosis, even surpassing human levels. Reinforcement learning theory fits well with the idea of cognitive radar in that both involve exploring the environment, sensing information about the surroundings and interacting with it, using it to make the best possible decisions to achieve the ultimate goal. For example, the most important feature of reinforcement learning is that decisions are learned through rewards, and the reward function can correspond to rating criteria in cognitive radar systems, such as the signal-to-jamming-noise ratio, amount of mutual information and probability of detection, among other criteria. While reinforcement learning seeks optimal solutions for specific objectives, cognitive radar requires the design of optimal transmitting waveforms to meet specific mission requirements to achieve optimal radar performance. Reinforcement learning requires consideration of the long-term nature of the strategy, and the constant game of cognitive radar against jamming is also a process of finding winning strategies in terms of long-term goals. Therefore, the use of reinforcement learning theory, which is highly compatible with cognitive radar and game theory, can help radar achieve interactive sensing and adaptive firing with the environment, improve the ability of intelligent sensing, processing and feedback of radar and realize the overall performance of radar in complex adversarial environments [4].

With the continuous research on arbitrary waveform transmitters, the maturity of cognitive radar theory and the wide application of artificial intelligence technology, exploring waveform design methods for intelligent anti-jamming of airborne radars based on cognitive radar ideas and artificial intelligence technology has become a feasible new anti-jamming approach for radars. The method can greatly improve the active anti-jamming performance of radars and better adapt to the complex environment of the future intelligent battlefield. A joint adaptive frequency hopping and pulse width allocation scheme based on reinforcement learning was proposed in the literature [24], and the effectiveness of the scheme was verified by simulation. In the literature [25], an energy-effective power control scheme based on reinforcement learning was proposed for detecting spoofing jamming in split-frequency array MIMO radars, which effectively improved the detection accuracy of spoofing jamming and saved radar energy. The literature [26] improved the agent of radar anti-jamming by designing anti-jamming strategies using reinforcement learning algorithms. The literature [27] proposed a reinforcement learning-based algorithm for cognitive multi-target detection in the presence of unknown jamming statistics, and experiments show that the radar target detection performance is significantly improved under harsh environmental conditions, such as low signal-to-noise ratio and dragging jamming.

In recent years, the field of reinforcement learning has made significant breakthroughs in many artificial intelligence tasks based on a combination of traditional theory and deep learning, giving rise to a new research area of deep reinforcement learning, which provides a viable way to solve complex scenario problems in the real world.

Deep Q network (DQN), one of the classical algorithms for deep reinforcement learning, is highly regarded by the academic community. To address the situation in which cognitive radars do not know the exact jamming model, Li K. et al. [28] used reinforcement learning algorithms to design a frequency hopping strategy based on frequency agile radar. Based on this, Li K. et al. [29] also proposed a DQN-based frequency agile (FA) radar strategy design method, which achieves radar anti-jamming with a high target detection probability. Then, they [30] applied deep reinforcement learning to the design of an anti-main-lobe jamming strategy for frequency shortcut radar and achieved intelligent selection of pulse carrier frequency. The literature [31] used deep Q networks and long- and short-term memory networks to generate frequency hopping strategies under a partially observable Markov decision process model to improve cognitive radar anti-jamming performance. To address the problem of coexistence between radar and communication systems, the literature [32,33] modelled the radar environment as a Markov decision process to predict the frequency band with the lowest jamming energy and successfully avoided jamming from communication systems. In the literature [34], a deep deterministic policy gradient cognitive design based on a control framework was proposed for the radar power allocation problem in multi-manoeuvre target tracking.

**Figure 1:**
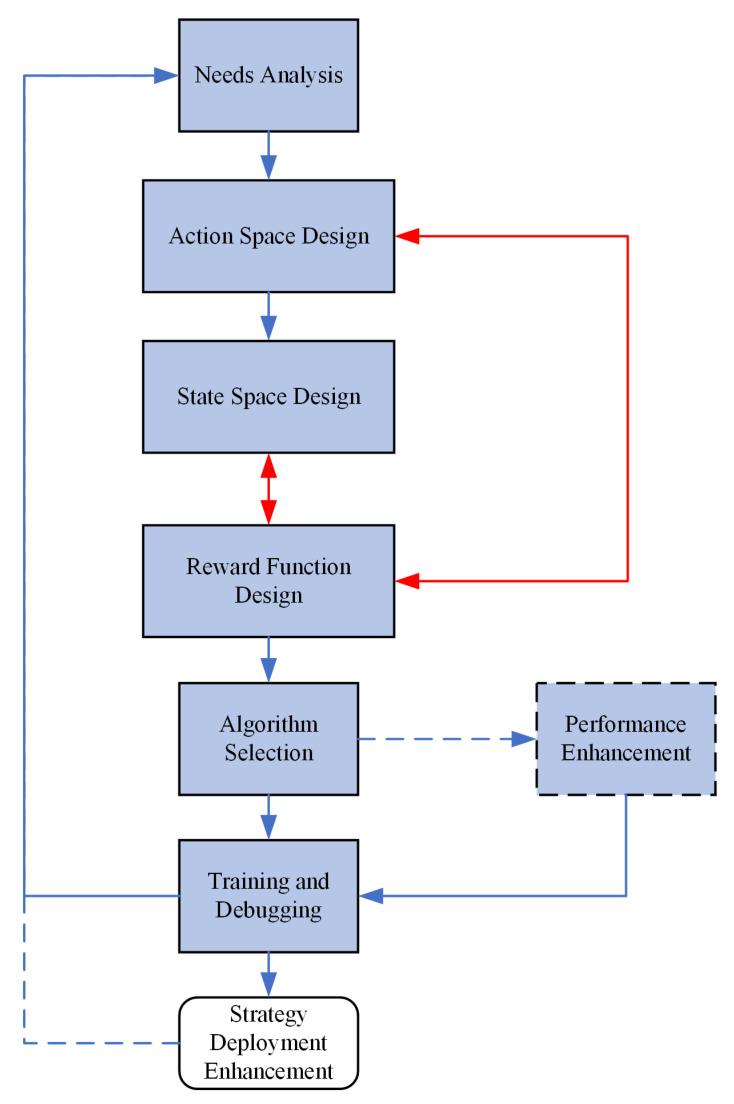
DRL algorithm landing process.

As a value-based algorithm, DQN can only solve finite and discrete action spaces. A series of improvements have been proposed in academia for the DQN algorithm and many research results have been published [35], such as distributional DQN, double DQN, duelling DQN, noisy DQN, etc. However, almost all DRL algorithms have different characteristics and limitations in their applications, as shown in Table 1. Therefore, when selecting DRL algorithms, the need for realistic scenarios and problems is fully considered. The goal of the development of radar countermeasure technology is to achieve intelligent radars and jammers with an observing environment and independent decision making. To solve the problem of radar countermeasures with complex strategies, deep learning techniques with good perception characteristics can be incorporated into reinforcement learning algorithms to better realize environment observation, learning calculation, decision making and feedback, forming a closed-loop process of “observation-action-feedback”, which can effectively improve the overall performance of the radar. Figure 1 shows the steps in the implementation of a rational application of the DRL algorithm to a practical problem, where the red line indicates co-design between modules and the dotted line indicates optional.

As shown in Figure 1, a DRL algorithm that wants to solve a practical task must go through the following steps: The first step is to conduct a requirements analysis to objectively assess the adaptability and performance improvement of the DRL for the task at hand and to identify the agent and environment in the task. Next, the core elements of the state space, action space and reward function are designed separately to complete the complete definition of the reinforcement learning problem. Then, a suitable DRL algorithm is selected with the task characteristics, and the policy model is obtained after training and debugging. If the performance of the policy is not satisfactory, the above process and algorithm details need to be continuously improved based on the results of the experiments and additional performance enhancement measures taken if necessary until a satisfactory policy is achieved. Ultimately, the trained policies are deployed in real application environments.

In summary, to address the problem of intelligent anti-jamming of airborne radars in complex electromagnetic environments, we should introduce cognitive radar ideas and establish Markov decision process (MDP) models to describe radar and target jamming countermeasures from the perspective of waveform design. Interaction between airborne radar and environmental information is determined via analyzing the spectral characteristics of target response, jamming signals, clutter response and airborne radar transmit waveforms. Using deep reinforcement learning algorithms, we study waveform design methods for airborne radars under jamming conditions and explore feasible ways for airborne radars with predictive and anti-intelligent jamming capabilities, laying a solid foundation for the realization of intelligent radars.

### 1.3. Main Contributions

In the first stage, we studied the anti-jamming problem of the airborne radar in a complex environment and proposed a radar anti-jamming waveform design method based on the strategy iteration method [43]. However, this method can only solve the problem of low-dimensional discrete spatial reinforcement learning, and it applies to situations where there are many influencing factors, high data dimension and a large scale in the actual radar confrontation process. To solve the above problems, we propose a deep reinforcement-learning-algorithm-based airborne radar waveform design method for clutter and jamming conditions. Firstly, a Markov decision process-based game model between airborne radar and jammer is developed to describe the actual operating environment of airborne radar containing information on target characteristics, clutter, noise and jamming signals, etc., by combining cognitive radar ideas. Secondly, we build deep neural networks to sense, analyze and learn from complex electromagnetic environments. Then, the optimal transmitted waveform for airborne radar is designed based on the D3QN algorithm. Finally, an iterative transformation method is used to synthesize the time domain radar signal for the optimal waveform strategy.

The main contributions of this paper are as follows.

- To address the problems of single and idealized airborne radar countermeasure scenario models, this paper investigates a Markov decision process (MDP)-based approach for modelling dynamic airborne radar countermeasure scenarios. The airborne radar electromagnetic environment under clutter and jamming conditions is investigated, and the dynamic countermeasure process between airborne radar and jamming is modelled using MDP. The MDP-based radar modelling approach allows for the analysis of the electromagnetic information present in realistic scenarios and the arbitrary design of countermeasure models containing a variety of influencing factors such as noise, clutter, the impulse response of the detected target, radar-transmitted signals and jamming signals. The method overcomes the limitations of traditional models and improves the flexibility, accuracy and predictability of the description of airborne radar operating scenarios, providing a viable way to realize the increasingly complex modelling of actual airborne radar operating scenarios.
- This paper proposes an intelligent waveform design method based on DRL to address the problem of anti-jamming of radar under clutter and jamming conditions. Radar-environment information interaction is determined by modelling the MDP for radar and jamming countermeasures. A radar frequency domain optimal anti-jamming waveform strategy generation method based on the D3QN algorithm is proposed. We build two deep neural networks to fit the state value function and action value function of the radar. The “overestimation” problem of the value function is solved using the fixed Q target method and the preferred experience replay method. To improve radar learning efficiency, prevent training overfitting, etc., the action value function network adopts a pairwise structure. Once the optimal frequency domain waveform strategy has been computationally selected, the ITM is used to generate the corresponding time domain signal with a constant envelope. The simulation results show that the radar transmit waveform designed by this method achieves intelligent anti-jamming of airborne radar while further improving the radar target detection probability compared to the conventional LFM signal, based on the radar waveform strategy generated by the strategy iterative method.
- Through extensive simulation experiments, the results show that the optimal waveform design method for airborne radar proposed in this paper significantly improves the anti-jamming performance and target detection performance of airborne radar, and outperforms traditional linear FM signals and previous research results based on reinforcement learning.

## 2. Airborne Radar Signal and MDP Model

### 2.1. Airborne Radar Signal Model

**Figure 2:**
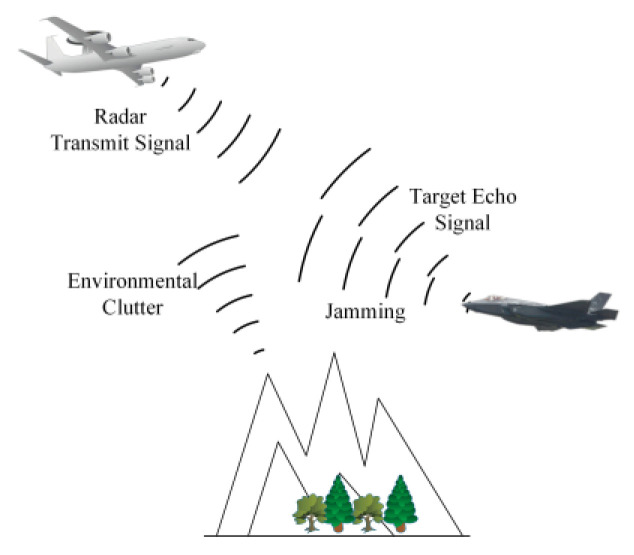
The airborne radar detection scene.

**Figure 3:**
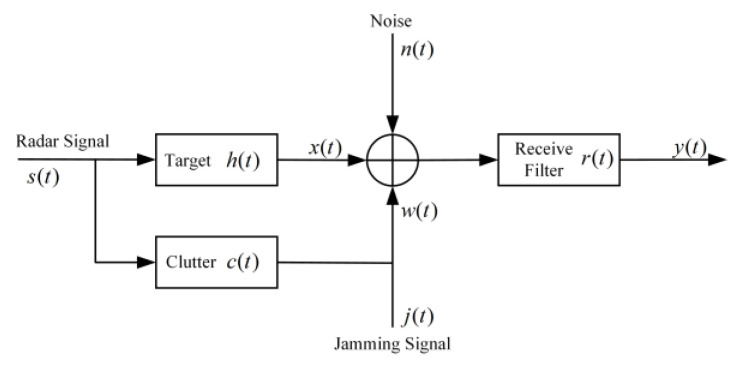
Airborne radar signal model.

Figure 2 shows the airborne radar detection scene. To establish the airborne radar signal model in a complex electromagnetic space, we need to fully consider the influence of radar-transmitted signals, enemy jamming signals, target echo, noise and all kinds of environmental clutter and other factors. Figure 3 shows the airborne radar signal model, where s(t) is the radar-transmitted signal, the Fourier transform is S(f), the signal bandwidth is W and the total power is $P_{S}$; j(t) is the jammer signal, the power spectral density is J(f) and the total power is $P_{J}$. The Fourier transforms of the target impulse response h(t) and the receive filter impulse response r(t) are H(f) and R(f), and h(t) is a time-limited random model. Information such as target impulse response and clutter response can be acquired during the airborne radar search phase. The clutter c(t) is a non-Gaussian random process, and the power spectral density $S_{c}(f)$ is not constant within W. The noise n(t) is a zero-mean Gaussian channel process with a power spectral density $S_{n}(f)$ that is not zero within W.

The expression of the signal y(t) at the output end of the radar receiver filter is as follows [44]:

(1) y(t)=r(t)∗(s(t)∗h(t)+s(t)∗c(t)+n(t)+j(t))

where “*” is the convolution operator. The radar signal components and jamming components are expressed as:

(2) $$y_{s}(t)=r(t)*(s(t)*h(t))$$

(3) $$y_{j}(t)=r(t)*(s(t)*c(t)+n(t)+j(t))$$

At the time $t_{0}$, the frequency domain expression of the signal-to-jamming-noise ratio (SJNR) is

(4) $${\left(SJNR\right)}_{t_{0}}=\frac{{\left|y_{s}(t_{0})\right|}^{2}}{E({\left|y_{j}(t_{0})\right|}^{2})}=\frac{{\left|\int_{-\infty }^{+\infty }R(f)H(f)S(f)e^{j2\pi ft_{0}}df\right|}^{2}}{\int_{-\infty }^{+\infty }{\left|R(f)\right|}^{2}(S_{c}(f){\left|S(f)\right|}^{2}+J(f)+S_{n}(f))df}$$

h(t) is a time-limited stochastic model, and the power spectral density can be replaced by energy spectrum variance [45].

(5) $$\sigma_{h}^{2}(f)=E({\left|H(f)-\mu_{h}(f)\right|}^{2})$$

Assuming that means is 0, substitute Equation (5) into Equation (4) and use Schwartz’s inequality to solve for Equation (6).

(6) $${\left(SJNR\right)}_{t_{0}}\leq \frac{\int_{-\infty }^{+\infty }{\left|R(f)\right|}^{2}(S_{c}(f){\left|S(f)\right|}^{2}J(f)+S_{n}(f))df\int_{-\infty }^{+\infty }\frac{\sigma_{h}^{2}(f){\left|S(f)\right|}^{2}}{S_{c}(f){\left|S(f)\right|}^{2}J(f)+S_{n}(f)}df}{\int_{-\infty }^{+\infty }{\left|R(f)\right|}^{2}(S_{c}(f){\left|S(f)\right|}^{2}+J(f)+S_{n}(f))df}$$

The equals sign holds under the condition that the SJNR takes the maximum value when and only when $R(f)=\frac{{\left[k\sigma_{h}(f)S(f)e^{j2\pi t_{0}}\right]}^{*}}{S_{c}(f){\left|S(f)\right|}^{2}+J(f)+S_{n}(f)}$ (k is an arbitrary constant). Assuming that the jammer can obtain the radar signal spectrum and adjust the jamming to the same band of the radar signal to achieve the maximum jamming effect, at this point, the following is obtained:

(7) $${\left(SJNR\right)}_{t_{0}}=\int_{-\infty }^{+\infty }\frac{\sigma_{h}^{2}(f){\left|S(f)\right|}^{2}}{S_{c}(f){\left|S(f)\right|}^{2}+J(f)+S_{n}(f)}df≃\frac{\Delta f}{W}\sum_{k=1}^{K}\frac{\sigma_{h}^{2}(f_{k}){\left|S(f_{k})\right|}^{2}}{S_{c}(f){\left|S(f_{k})\right|}^{2}+J(f_{k})+S_{n}(f_{k})}$$

where K is the number of frequency samples, and Δf is the frequency sampling interval, KΔf=W.

### 2.2. MDP Model

**Figure 4:**
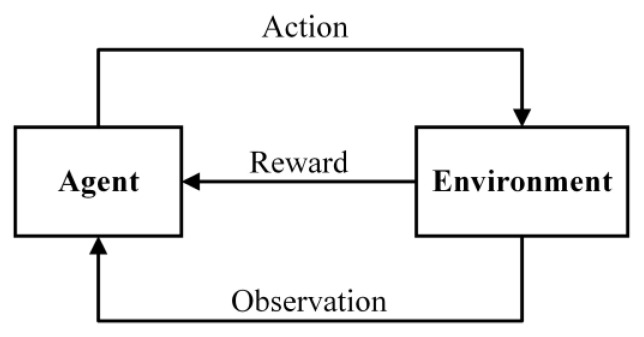
Reinforcement learning model.

Agents are actionable entities for reinforcement learning. For self-driving cars, the environment is the current road conditions; for Go, the status is the current game. At each moment, the agent and environment have their state, such as the current position and speed of the car, the vehicle and pedestrian conditions on the road. The agent determines an action based on the current state and performs that action. It then moves on to the next state with the environment, and the system gives it a feedback value, rewarding or punishing the action, and forcing the agent to perform the desired action. As shown in Figure 4.

Reinforcement learning is a class of methods that solve such decision-making problems. The algorithm learns a mapping function called the strategy function through sample learning, whose input is the environment information at the current moment, and the output is the action to be performed, in this case a=π(s), where s is the state, a is the action to be performed, and the state and action are from the state set and the action set, respectively. Actions and states can be discrete or continuous. For the former, the set of actions and states is finite, and for the latter, it is an infinite set. The goal of acting is to achieve a certain purpose, such as driving safely in an unmanned car, winning this Go game and modelling this with a reward function.

The problems to be solved by reinforcement learning can be abstracted into the MDP. Markov processes are characterized by the fact that the state of the system at the next moment is determined by the state of the current moment, regardless of the earlier moment. Unlike the Markov process, in MDP, the agent can perform actions that change the state of itself and the environment and receive a punishment or reward.

The MDP model can be described by a five-tuple $\left\{S,A,P,R,\gamma \right\}$, where S is the state space, A is the action space, P is the state transfer probability, R is the payoff function, and γ is the discount factor [46]. The state space S is the set of all the factors involved in the confrontation scenario; the action space A is the set of all possible behaviours. The state transfer probability function P(s,a,s') is the probability of the agent taking action a from the state s to transition to the state s'. The reward function R(s,a,s') is the average value of the cumulative rewards for the agent performing action a transition to a state s' in the state s. A positive reward value is rewarded for the selected action and a negative one is penalized. To ensure the convergence of the algorithm, a discount factor γ∈[0,1] is set. When γ tends to 0, the agent tends to obtain immediate rewards; when γ tends to 1, the agent prefers to obtain long-term gains.

## 3. MDP-Based Radar Countermeasure Environment Modelling

### 3.1. Radar Action, Status and Reward Design

**Figure 5:**
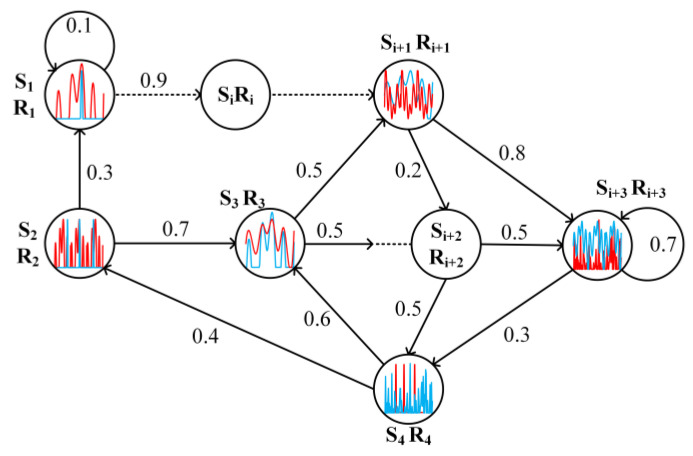
The radar countermeasure process based on markov decision process (MDP).

The radar transmits signals to detect the target and the jammer performs jamming on the received radar signals. Radar for the electromagnetic space of jamming signals, environmental clutter and the characteristics of the target being detected integrates this information to design a new radar-transmitted signal to complete the anti-jamming and target detection tasks. The waveform changes in the process of radar-jamming confrontation are Markovian, and the radar confrontation environment can be modelled as an MDP model to achieve intelligent anti-jamming waveform design for airborne radars by interacting with environmental information, such as environmental noise, clutter and jamming signals. Figure 5 briefly describes the MDP-based radar countermeasure process, where blue represents the radar signal, the red waveform represents the jamming signal, $S_{i}$ is the current state, $R_{i}$ is the reward for the current state, and the number on the arrow indicates the transfer probability between states.

The radar signal s(t) and the jamming signal j(t) are equally divided into M sub-bands in the frequency domain, and the sub-band power is equally divided into N parts; i.e., the individual signal is modelled as an array consisting of M numbers, as shown in Equation (8).

(8) $$s(t),j(t)\Rightarrow [f_{1},f_{2},\cdots ,f_{M}],f_{i}\in [0,1,\cdots ,N]$$

All jamming signals form the state space S, which is defined as

(9) $$S=(s_{1},s_{2},\cdots ,s_{{\left(N+1\right)}^{M}})$$

where the subscript ${\left(N+1\right)}^{M}$ denotes the size of the state space S, and $\alpha_{i}\in [0,1,\cdots ,N],i\in [1,2,\cdots ,M]$ denotes the signal power allocation size of the sub-band i of a single state $s_{t}$ at the moment t, which can be expressed as

(10) $$s_{t}=[\alpha_{1},\alpha_{2},\cdots ,\alpha_{M}]$$

Similarly, all radar signals form the action space A, which is defined as

(11) $$A=(a_{1},a_{2},\cdots ,a_{{\left(N+1\right)}^{M}})$$

where the subscript ${\left(N+1\right)}^{M}$ denotes the action space A size, and $\beta_{i}\in [0,1,\cdots ,N],i\in [1,2,\cdots ,M]$ denotes the signal power allocation size of the sub-band i of action $a_{t}$ taken at the moment t, which can be expressed as

(12) $$a_{t}=[\beta_{1},\beta_{2},\cdots ,\beta_{M}]$$

The reward is the key factor that affects the good or bad decision. The SJNR of the radar signal is used as the action reward of the intelligent body decision, and the reward value size is proportional to the radar signal SJNR; the larger the SJNR, the larger the reward.

(13) $$\mathrm{Reward}\propto SINR≃\frac{\Delta f}{W}\sum_{k=1}^{K}\frac{\sigma_{h}^{2}(f_{k}){\left|S(f_{k})\right|}^{2}}{S_{c}(f){\left|S(f_{k})\right|}^{2}+J(f_{k})+S_{n}(f_{k})}$$

### 3.2. Confrontation Model Key Parameter Settings

Setting M=5, N=5, the airborne radar and jammer MDP game model uses an array of five digits to represent the frequency domain energy distribution state of the radar signal s(t) and jammer signal j(t). The signal frequency domain is divided into five sub-bands, and the sub-band power is divided into five equal parts with a discount factor γ=0.9. The specific parameter settings are shown in Table 2.

## 4. D3QN-Based Strategy Generation for Optimal Radar Anti-Jamming

In this paper, the MDP model is established for the radar anti-jamming environment under clutter and jamming conditions. Firstly, the deep neural network is trained to realize the perceptual interaction with complex electromagnetic environment factors such as clutter, noise and jamming. Second, the SJNR of the radar signal is set as the reward function by combining the impulse response of the detected target acquired in advance. Then, the frequency domain energy optimal strategy of the radar anti-jamming signal is obtained based on the D3QN algorithm, and its time domain constant mode signal is synthesized to realize the intelligent anti-jamming waveform design for airborne radar.

**Figure 6:**
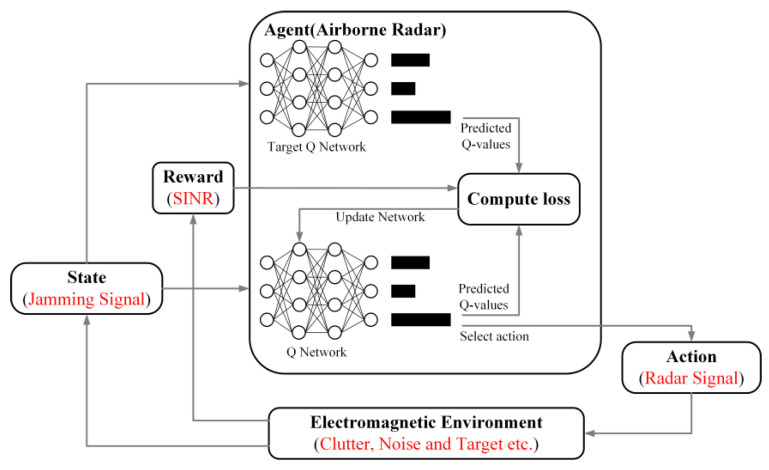
Block diagram of the radar intelligent anti-jamming based on the duelling double deep Q network (D3QN).

The principle of D3QN-based radar intelligent anti-jamming is shown in Figure 6. The radar is regarded as the agent, the jamming signal in the electromagnetic space as the state information, the radar-transmitted signal as the action and the SJNR of the radar signal as the reward function. This is achieved by firstly continuously calculating the reward values for taking different actions in different states, and storing the corresponding state, action and reward information for training the neural network. Secondly, the neural network analyzes, calculates and selects the radar anti-jamming strategy with the highest Q value. Finally, the selected optimal anti-jamming strategy is output to the radar to synthesize the time domain signal and transmit it.

### 4.1. Fixed Q Targets

Due to the max operation, the value function has an overestimation problem at each point of the value, and an uneven amount of overestimation leads to a suboptimal solution, so a method for fixing the Q target is needed.

First, an evaluation network and a target network are created. A target network with fixed parameters $\omega_{t}$ is used to estimate the time difference target. Second, a function is created to obtain the evaluation network parameters and replicate them to the target network. After performing N updates, the parameters $\omega_{e}$ of the evaluation network are periodically copied to the target network to update the parameters $\omega_{t}$ of the target network.

(14) $$\Delta \omega =\alpha [(R+\gamma \max_{a}\overset{\wedge }{Q}(s^{\prime },a,\omega_{t}))-\overset{\wedge }{Q}(s,a,\omega_{e})]\nabla_{w}\overset{\wedge }{Q}(s,a,\omega_{e})$$

(15) $$\mathrm{At}\;\mathrm{every}\;N\;\mathrm{step}:\;\omega_{t}\leftarrow \omega_{e}$$

Then, the evaluation network is used to obtain the action corresponding to the optimal action value in the state $s_{t+1}$, and the target network is used to calculate the action value of this action to obtain the target value. The interaction of the two networks effectively avoids the problem of the “overestimation” of the algorithm.

(16) $$y_{t}=r_{t+1}+\gamma q(s_{t+1},\arg\max_{a}q(s_{t+1},a;\omega_{e});\omega_{t})$$

### 4.2. Prioritized Experience Replay

**Figure 7:**
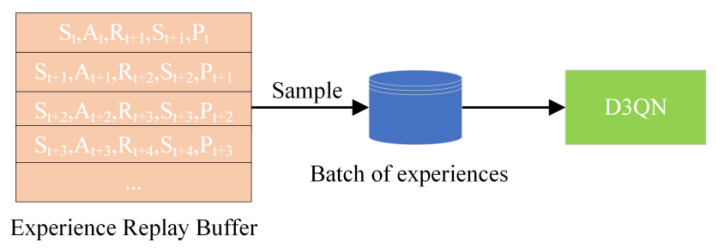
Schematic diagram of the empirical replay process.

To help the radar fully learn and adapt to the current electromagnetic countermeasure environment and to improve the speed of environmental information acquisition, an empirical replay method is used. First, the data, such as the action, a, selected by the radar, the new jamming state received, $s^{\prime }$, the calculated reward value, R, and whether the interaction is in the terminated state, done, in each jamming state, s, during the interaction between the radar and the complex electromagnetic environment, are recorded as $\left\{S,S^{\prime },A,R,\;done\right\}$, and all are stored in the replay buffer D. Second, when the number of data reaches a set batch size, a batch of data is evenly and randomly selected and put into the neural network for training. Then, after training, the radar continues to interact with the electromagnetic environment and repeats the operation. A batch size of data is sampled uniformly and randomly each time to train the neural network, which improves data utilization and reduces the overfitting problem arising from training. The experience replay process is shown in Figure 7.

However, the uniform random sampling of data during the neural network training process suffers from insufficient learning of important experiences. To avoid the possibility that more important radar countermeasure experiences may occur less frequently than others, a priority experience replay method is adopted so that important experiences have a high probability of being replayed, thus greatly improving the efficiency of the utilization and learning of radar countermeasure data [40].

The radar adversarial process is a model-free reinforcement learning, which requires the use of the concept of time difference error (TD error) to represent the difference between the current Q value and the target Q value as a measure of the importance of each set of radar training data. If the TD error is larger, it means that there is still much room for improvement in prediction accuracy, and the more intelligent the body updates, the more this sample needs to be learned, i.e., the higher the priority p is. Let the TD error at the sample i be $\sigma_{i}$; then, the sampling probability at the sample is [40].

(17) $$P(i)=\frac{p_{i}^{a}}{\sum_{k}p_{k}^{a}}$$

where $p_{i}$ denotes the priority level and both are greater than 0, α determines how many heavyweights to use, and if α=0, no importance sampling is used, that is, uniform random sampling. Priority $p_{i}$ is defined by the proportional prioritization method, as shown in Equation (18).

(18) $$p_{i}=\left|\delta_{i}\right|+e$$

where $\left|\delta_{i}\right|$ is the magnitude of the TD error and e is a small normal number, ensuring that some special edge examples with a TD error of 0 can also be extracted.

With probability distribution sampling with priority playback, the action value estimate is biased because the sample distribution and the action value function distribution are two completely different distributions. To correct this deviation, it is necessary to multiply it by an importance sampling coefficient, $\omega_{i}$ [40]:

(19) $$\omega_{i}={\left(\frac{1}{N}\cdot \frac{1}{P(i)}\right)}^{\beta }$$

These weights are added to the Q network update using $\omega_{i}\delta_{i}$.

### 4.3. Value Function V and Dominance Function A

**Figure 8:**
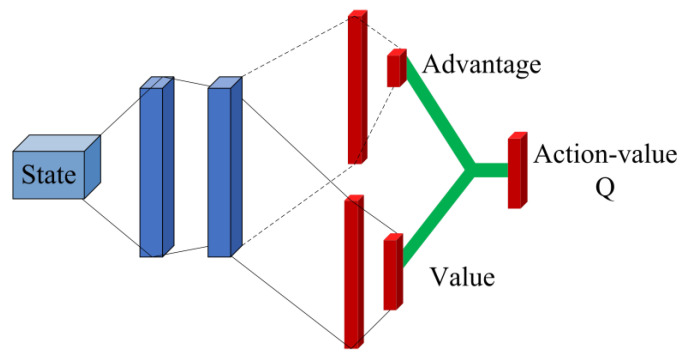
Paired network structure.

In the radar intelligent anti-jamming training, to improve the radar learning efficiency and prevent training overfitting and other situations, the action value function network outputs the state value function and the dominance function, respectively, in the hidden layer in the middle of the network by adopting a dyadic network structure using two fully connected sequences. This is shown in Figure 8 [41].

The advantage function A(s,a) represents the advantage of taking this action in the current state, and the advantage here is the advantage of the action value function over the value function of the current state, as shown in Equation (20).

(20) A(s,a)=Q(s,a)−V(s)

where the action value function Q(s,a) is the value corresponding to a single action. V(s) is the action value function of all possible actions in the state s multiplied by the probability sum of taking the action; that is, the value function V(s) is the average of all the action value functions in that state concerning the probability of action, as shown in Equation (21).

(21) $$V_{\pi }(s)=\sum_{a\in A}\pi (a|s)q_{\pi }(s,a)$$

By calculating the advantage function, you can compare the average size of the action value of the current radar anti-jamming strategy relative to the action value function. If the advantage function is greater than 0, the action is better than the average action, and vice versa; the current action is not as good as the average action.

Thus, the Q value corresponding to each action can be calculated using Equation (22), and the output of the action value function network is:

(22) $$Q(s,a;\theta ,\alpha ,\beta )=V(s;\theta ,\beta )+(A(s,a;\theta ,\alpha )-\frac{1}{A}\sum_{a^{\prime }}A(s,a^{\prime };\theta ,\alpha ))$$

Among them, θ is a commonly used network parameter, α is the advantage function network parameter, β is the state value function network parameter, and $\frac{1}{A}\sum_{a^{\prime }}A(s,a^{\prime };\theta ,\alpha )$ is a centralized processing of the advantage function to ensure that there will be zero advantage under a certain action.

### 4.4. Design Flow of Optimal Anti-Jamming Strategy for Radar Based on the D3QN Algorithm

In summary, combined with the actual countermeasure scenario, the airborne radar anti-jamming problem is transformed into a reinforcement learning problem, the countermeasure model between radar and target is established, and the optimal anti-jamming strategy is obtained by describing the electromagnetic environment and training the neural network based on the D3QN algorithm with the following algorithm steps (Algorithm 1).

**Algorithm 1:** Airborne radar anti-jamming strategy design algorithm based on D3QN.
Initializes the current Q network parameter θ, and initializes the target $Q^{\prime }$ network parameter $\theta^{\prime }$. The Q network parameters are assigned to the $Q^{\prime }$ network, $\theta \to \theta^{\prime }$. The total number of iterations T, discount factor γ, exploration rate ε, target Q network parameter update frequency P, and the number of samples sampled at a time m are determined.Initialize the replay buffer D.For t=1 to T:

1)Initialize the environment and obtain the state S (i.e., jamming signal), R=0,done=False.2)while True:
a)Based on the state ϕ(S) acquisition, input the current Q network, calculate the Q value corresponding to each action (i.e., radar signal), and use the ε -greedy algorithm to select the action A corresponding to the current state S.b)Perform the action A, obtain new status $S^{\prime }$ and rewards R, whether the anti-jamming process is in the end state donec)Save 5 elements $\left\{S,S^{\prime },A,R,\;done\right\}$ into D.d)if done:breake)Randomly sample m samples from D, $\left\{S,{S^{\prime }}_{j},R_{j},A_{j},{\mathrm{done}}_{j}\right\}$, j,1,2,3,4,…,m. Calculate $y_{j}$ of the current Q network $y_{j}=R_{j^{\prime }}+\gamma Q^{\prime }(\phi ({S^{\prime }}_{j}),\arg\max_{a^{\prime }}Q(\phi ({S^{\prime }}_{j}),a;\theta );\theta^{\prime })$.f)Use the mean squared loss function $(\frac{1}{m}){\sum_{r=1}^{n}\left(y_{j}-Q(\phi (S_{j}),A_{j},\theta )\right)}^{2}$, calculate loss, and backpropagate update parameter θ.g)if $t\%p=0:\theta \to \theta^{\prime }$h)$S^{\prime }=S$



### 4.5. Performance Indicators

The radar detection problem in this paper can be defined as a hypothesis testing problem [47]. By solving the classical Neyman–Pearson theorem, the radar target detection probability $P_{D}$ is obtained as

(23) $$P_{D}=Q(Q^{-1}(P_{FA})-\sqrt{d^{2}})$$

where $P_{FA}$ is the probability of a false alarm, $P_{FA}=Q(\tau )=\int_{\tau }^{\infty }\frac{1}{\sqrt{2\pi }}\exp(-\frac{1}{2}t^{2})dt$, τ is the radar detection threshold, and $d^{2}$ is the offset coefficient, which is the SJNR in this model. The detection performance of such detectors is completely determined by the offset coefficient, so by calculating the SJNR, the relationship between radar waveform and target detection probability can be established.

## 5. Simulation Results and Analysis

**Figure 9:**
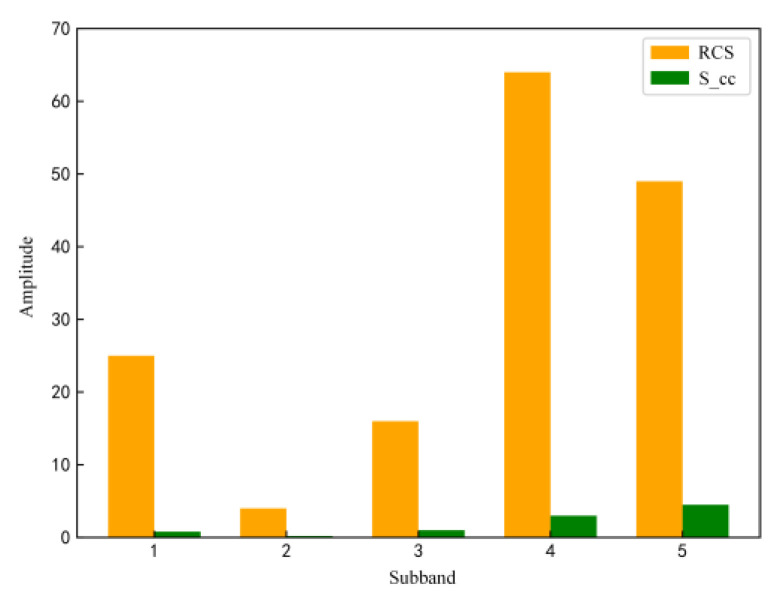
Environmental clutter and target impulse response.

The simulation was carried out based on foreign airborne radar setting parameters such as operating band, centre frequency and signal bandwidth, as well as target flight speed, target impulse response and environmental clutter information, as shown in Table 3. The environmental information is shown in Figure 9. Yellow indicates the target impulse response information and green indicates the environmental clutter information. The signal power of the sub-bands in all result maps is in per cent.

### 5.1. Radar and Jamming Game Strategy Generation

**Figure 10:**
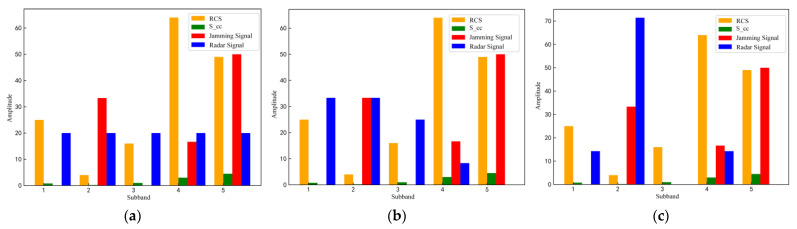
Three radar-transmitted signals under jamming conditions. (**a**) linear frequency modulation (LFM) signal; (**b**) anti-jamming signal generated based on policy iteration method; (**c**) radar optima anti-jamming signal generated based on D3QN.

During the confrontation between radar and jamming, the radar generates the optimal anti-jamming waveform frequency domain strategy by interacting with the environment using the D3QN algorithm. To verify the effectiveness of the method in this paper, experimental comparisons were made between the conventional LFM signal of the radar and the frequency domain anti-jamming strategy generated by the team’s previous iterative method based on the classical reinforcement learning strategy under the same experimental conditions, respectively, and the results are shown in Figure 10.

As can be seen from Figure 10a, because the LFM signal has a large temporal bandwidth product, and the top undulation of the amplitude–frequency characteristics of the signal gradually decreases with the increase in the temporal bandwidth product and approaches rectangular shape, but the signal power is uniformly distributed in five sub-bands, which cannot be used for radar anti-jamming according to the current electromagnetic environment information and target characteristics. From Figure 10b,c, it can be seen that both the radar anti-jamming strategy generated based on the strategy iteration method and the radar anti-jamming strategy generated based on the D3QN algorithm in this paper can respond to the clutter, jamming and target characteristics to some extent to achieve the anti-jamming effect. As shown in Figure 10b, the radar anti-jamming strategy based on the strategy iterative method mainly distributes the radar signal power in sub-frequency bands 1 and 3 with no jamming signal and strong target impulse response, indicating that the strategy can respond to environmental information to a certain extent. In Figure 10c, under the same environmental conditions, the radar optimal anti-jamming strategy obtained by the proposed method is more sensitive to the clutter, jamming signal and the impulse response of the detected target, and the strategy distributes most of the radar signal power in the sub-frequency band with the low power of the clutter and jamming signal and the sub-frequency band 1, 2 and 4 where the target impulse response is strong, to obtain a higher radar SJNR and the detected target information.

### 5.2. Performance Comparison

**Figure 11:**
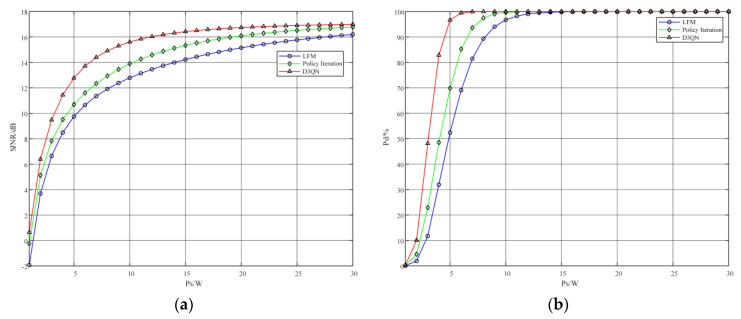
Performance of different signals when radar power changes. (**a**) the signal-to-jamming plus noise ratio (SJNR) of different transmitted signals; (**b**) detection probability of different transmitted signals.

The SJNR is an important parameter for measuring the performance of radar target detection. To verify the performance of the D3QN-based algorithm for airborne radar anti-jamming strategy design, the optimal anti-jamming waveform strategy designed in this paper is compared with the LFM signal and the anti-jamming signal designed by the classical strategy iteration method in reinforcement learning, and the target detection performance of the optimal waveform strategy is analyzed. The radar receiver SJNR and radar target detection probability are calculated by Equations (7) and (23), respectively, to compare and analyze the optimal strategy performance, and the simulation results are shown in Figure 11.

#### 5.2.1. Performance Analysis When the Total Target Disturbance Power Pb Is a Constant Value

The simulation results in Figure 11 were achieved under experimental conditions where the jamming was a fixed total power of 10 W and the transmit power of the radar signal was $P_{s}=1-30W$. The variation in the SJNR and target detection probability with the total radar signal power for the three radar waveform strategies is shown in Figure 11.

The simulation results in Figure 11 were achieved under the experimental conditions where the jamming was a fixed power of 10 W and the transmit power of the radar signal was increased from 1 W to 30 W. The blue line in the figure indicates the LFM signal, and the green and red lines indicate the waveform strategy generated by the strategy iterative method and the optimal strategy generated by the D3QN algorithm based on this paper, respectively. From Figure 11a, it can be seen that the SJNR of all three different signals keeps increasing as the radar signal power increases, and the anti-jamming strategy generated by the D3QN algorithm based on the D3QN algorithm is significantly better than the other two strategies, and the SJNR of the anti-jamming strategy generated by the strategy iteration method based on the reinforcement learning tradition is also higher than that of the radar LFM signal. At the radar signal power of 10 W, the SJNR of the optimal strategy generated by this paper is up to 15.8 dB, which is 1.8 dB and 3.0 dB higher than that of the signal generated by the strategy iterative method and the LFM signal, respectively. Similarly, it can be seen from Figure 11b that among the three different signals, the signal generated by this method has the highest target detection probability, the signal generated by the MDP-based strategy iterative method has the second highest probability, and the LFM signal has the lowest detection probability. The LFM signal has the lowest detection probability. At the radar signal power of 5 W, the target detection probability of the optimal strategy generated by this paper is up to 97%, which is 27% and 44% higher compared to the signal generated by the strategy iterative method and the LFM signal, respectively.

#### 5.2.2. Performance Analysis When the Total Radar Signal Power Ps Is a Constant Value

**Figure 12:**
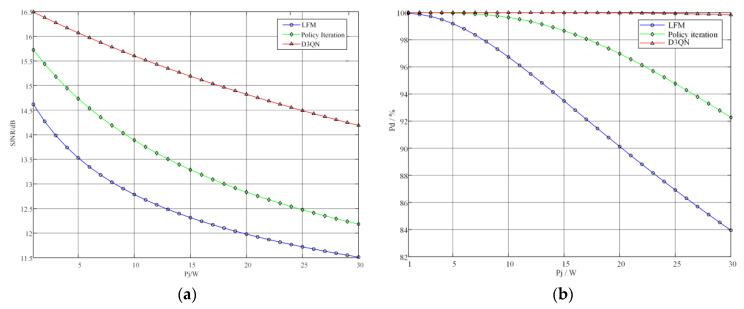
The performance of different signals when the jamming power changes. (**a**) SJNR of different transmitted signals; (**b**) detection probability of different transmitted signals.

The simulation results in Figure 12 were achieved under experimental conditions where the total power of the radar transmit signal was fixed at 10 W and the total target jamming power was $P_{j}=1-30W$. The SJNR and target detection probability of the three radar waveform strategies with the variation in the total jamming power are shown in Figure 12.

From Figure 12a, it can be seen that the SJNR of all three radar transmit signals decreases continuously as the jamming signal power increases, but the SJNR of the anti-jamming waveform strategy generated by the D3QN algorithm decreases significantly less than the other two strategies, and the anti-jamming strategy based on the strategy iteration algorithm also significantly outperforms the LFM signal. Similarly, as can be seen from Figure 12b, the target detection probability of the three radar signals decreases as the jamming power increases, but the optimal radar strategy obtained in this chapter has a lower target detection probability than the other two strategies, being 2.01 dB and 2.68 dB higher, respectively. Until the jamming power increases to 30 W, the target detection probability of this optimal strategy is still 99.84%, which is 7.57% and 15.89% higher than the signals generated by the iterative strategy method and the LFM signal.

#### 5.2.3. Performance Analysis When the Total Radar Signal Power Ps Is a Constant Value

**Figure 13:**
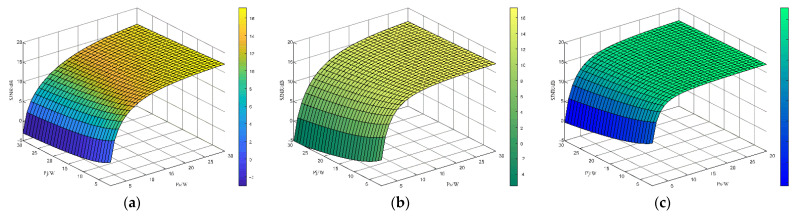
SJNR of the three signals under the conditions of jamming power and radar power variation. (**a**) Variation in LFM signal SJNR; (**b**) SJNR change graph of the anti-jamming signal generated based on strategy iteration method; (**c**) variation diagram of optimal anti-jamming signal SJNR generated based on D3QN algorithm.

For more visualization, a 3D plot reflecting the variation in the SJNR with $P_{s}=1-30W$ and $P_{j}=1-30W$ for the three radar waveform strategies in the clutter and jamming environment is shown in Figure 13.

Figure 13 shows the changes in the SJNR of the three signals, including the LFM signal, the radar anti-jam signal generated based on the strategy iterative method and the radar signal designed by the proposed method, when both the jamming power and the radar power increase from 1 W to 30 W. It can be seen that the SJNR of all three signals increases with the increase in radar power and decreases with the increase in jamming power. The simulation shows that the SJNR of the radar anti-jamming waveform generated by the method in this paper is the largest and has better anti-jamming performance when the jamming power is the largest (30 W) and the radar signal power is the smallest (1 W) under the same conditions.

### 5.3. Time Domain Waveform Synthesis

To better apply this method to practical radar detection systems and improve target detection performance, it is necessary to generate the time-domain-transmitted signals of the optimal waveform strategy in the radar frequency domain. There are many methods to obtain the time domain characteristics of the generated signal, and the simplest method is the direct inverse fast Fourier transform (IFFT) method, which performs an IFFT on the optimal amplitude spectrum and then normalizes the transformed signal in terms of amplitude. However, the time domain signal synthesized by this method differs significantly from the optimal strategy [48]. The fixed-phase technique is the conventional method for synthesizing nonlinear frequency-modulated signals and uses Newton’s method to calculate the numerical solution, which is complicated to derive. Jackson et al. [49] used the iterative transform method (ITM) to generate a constant envelope time domain signal with the best spectral fit. Therefore, in this section, ITM is used to fit the time domain signal with the optimal strategy in the frequency domain. The time domain form and spectral fitting results of the optimal radar waveform strategy designed based on the D3QN algorithm are as follows.

**Figure 14:**
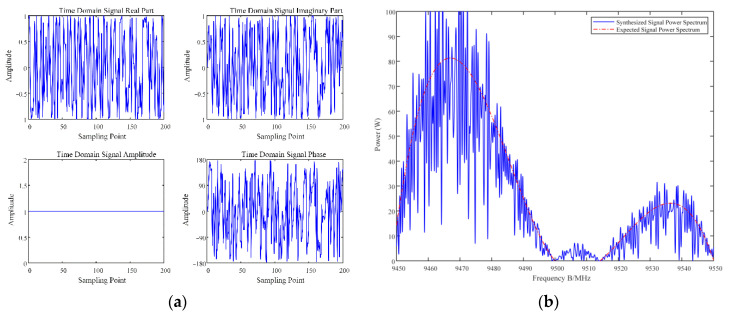
Time domain waveforms of the optimal radar anti-jamming strategy. (**a**) Magnitude and phase of the synthetic radar signal; (**b**) spectrum fitting results of the radar signal.

The radar time domain signal is synthesized according to the radar optimal anti-jamming frequency domain strategy in Figure 10c, and its frequency domain characteristics are verified, and the results are shown in Figure 14. Among them, Figure 14a depicts the real part, imaginary part, amplitude spectrum and phase spectrum of the synthesized time domain signal; Figure 14b shows the results of the frequency domain characteristics of the verified time domain waveform; the red dashed line is the optimal frequency domain strategy designed by the method in this paper, and the black solid line indicates the spectrum of the synthesized time domain signal by ITM. It can be seen that the synthesized time domain signal better achieves the frequency domain characteristics of the optimal strategy and has low interception performance, showing behaviours such as constant envelope and anti-jamming.

## 6. Conclusions

Airborne radars play a vital role in modern national defence by carrying out important tasks such as target search, detection, identification, tracking and imaging. With the rapid development of electronic countermeasure technology, the electromagnetic signals emitted by numerous electronic devices on the battlefield, enemy intelligent jamming signals and complex terrain clutter pose a serious threat to the survival and performance of airborne radars. Therefore, it is necessary to conduct research on anti-jamming technology for airborne radars in complex environments. In recent years, artificial intelligence algorithms have been widely used to provide a viable path for the implementation of cognitive radar. Cognitive radar waveform design is a main research area, involving modelling complex real-world operating environments. How to realize the interactive sensing of radar and electromagnetic environment information, make full use of target characteristics and jamming signal spectrum characteristics, design radar-transmitted waveforms in real time to adapt to complex environments and detected targets and achieve good anti-jamming while improving radar target detection performance is the focus of current research.

### 6.1. Article Work Summary

This paper outlines the current research status of airborne radar anti-jamming technology with research on deep-reinforcement-learning-based radar waveform design in complex environments and a focus on solving the dynamic confrontation problem between radar and jamming in electronic warfare, fundamentally improving radar’s active anti-jamming capability, enhancing radar adaptability in complex environments and laying the foundation for the development of radar confrontation. The main contributions of this work are as follows:

1. In response to the problems of single and idealized radar countermeasure scenario models in traditional anti-jamming technology research, we study a dynamic radar countermeasure scenario modelling method based on the Markov decision process. The MDP model overcomes the limitations of traditional models, improves the accuracy and predictability of the actual radar operating environment and provides a model basis for radar waveform design research. The MDP model overcomes the limitations of traditional models, improves the accuracy and prediction of the actual radar operating environment and lays the model foundation for post-radar waveform design research, further promoting the development and application of cognitive radar.
2. To address the problem of dynamic countermeasures between airborne radar and jamming in complex environments, we propose a deep-reinforcement-learning-based anti-jamming waveform design method for airborne radar under clutter and jamming conditions from a waveform design perspective and build a dynamic countermeasure model for airborne radar and jamming in complex environments based on MDP. We construct two deep neural networks for training learning of the neural networks by iteratively computing the reward values to the adversarial data using different radar waveforms in different jamming states. We recommend a design technique for the optimal anti-jamming waveform strategy of airborne radar based on the D3QN algorithm and synthesize the optimal time domain transmitted signal by iterative transformation method. It has certain reference significance for the realization of radar intelligent confrontation technology in electronic warfare and has laid a theoretical foundation for the realization of cognitive radar.

### 6.2. Future Research Perspectives

This paper mainly aims to investigate the design of anti-jamming waveforms for airborne radars in complex environments based on deep reinforcement learning, drawing on cognitive radar ideas. The paper has carried out some work at the basic theoretical level and obtained some preliminary research results, which should be followed up by research on the following aspects and shortcomings. The main areas include the following:

1. The waveform design problem for complex environments has focused on the waveform design problem for electromagnetic information environments containing clutter, noise and jamming, while the waveform design for the non-smooth and non-uniform actual combat environments faced by airborne radars has still not been considered. Therefore, subsequent work should fully investigate waveform design methods for airborne radars in various non-smooth, non-uniform electromagnetic environments, such as clutter and new types of jamming signals, and discuss the performance of waveform design technology methods in a variety of complex environments, which is of great significance for improving the anti-jamming performance of airborne radars in actual combat.
2. In response to traditional anti-principal flap jamming techniques’ difficulty coping with diverse and dexterous new types of jamming, we investigate a deep-reinforcement-learning-based approach to airborne radar waveform design. When building a dynamic countermeasure model between radar and jamming based on a Markov decision process, a discrete signal form and a reward function design based on a single performance indicator of the SJNR are mainly used, without considering continuous state and action representations, and a reward function setting that combines multiple performance indicators. The future development trend of radar must be multi-functional and intelligent, and in the subsequent research, attempts should be made to establish a continuous state and action space, which is conducive to the application of airborne radar intelligent waveform design technology in practice; the use of multiple radar performance indicators to combine the design of reward functions is of great significance to the development of multi-functional radar waveform design.
3. To address the difficulty of the practical application of theoretical waveform design methods, we focus on the design of frequency domain optimal anti-jamming waveform strategies based on artificial intelligence algorithms, using an iterative transformation method to synthesize the corresponding constant envelope time domain signal. However, only the real, imaginary, amplitude and phase information of the time domain signal is obtained, and no actual transmittable time domain waveform is found. Most of the current radar waveform design studies do not consider the actual emission of the designed waveform. On the one hand, this is because the modelling of the problem deviates significantly from the actual environment when considering waveform design methods, making it difficult to apply to real-world scenarios. On the other hand, the hardware is limited by the production process of electronic equipment, and current radar transmitters are not yet able to meet the demand for transmitting arbitrary waveforms. Therefore, to ensure that radar waveform design research results can be used in practical equipment and truly improve airborne radar performance, future radar waveform design research should be closely integrated with practical application scenarios and equipment, so as to lay a solid foundation for the successful implementation of airborne radar waveform design and the smooth development of new system radars.

## Figures and Tables

**Table 1:** Comparison of methods and results of research in related work.

Classification by DRL Algorithm	DRL Algorithm	Brief Description	References
Value-based	Q learning (1992.05)	Q learning uses Q Table to store the value of each pair of state actions. When the state and action space are high-dimensional or continuous, it is not realistic to use Q Table.	Watkins et al. [36]
	Deep Q Network (DQN,2013.12)	DQN can directly use raw high-dimensional sensor data for reinforcement learning, and training using stochastic gradient descent and the training process is more stable.	Mnih et al. [37]
	Distributional DQN (2015.08)	In DQN, the expected estimate of the state-action value Q of the network output ignores a lot of information. A deep reinforcement learning model modelled from a distributed perspective can obtain more useful information and obtain better and more stable results.	Bellemare et al. [38]
	Double DQN (2015.12)	In the original DQN, there will be a situation where the Q value is overestimated. To decouple the action selection and the value estimate, when calculating the actual value of Q in double DQN, the action selection is obtained by the evaluation network, while the value estimate is obtained by the target network.	Hasselt et al. [39]
	Prioritized Experience Replay (2016.02)	In the experience pool of traditional DQNs, the data selected for batch training are random, without taking into account the priority relationship of the samples. This method gives each sample a priority and samples according to the priority of the sample.	Schaul et al. [40]
	Duelling DQN (2016.04)	In the original DQN, the neural network directly outputs the Q value of each action, while the Q value of each action of the duelling DQN is determined by the state value V and the dominance function A, that is, Q = V + A.	Wang et al. [41]
	Noisy DQN (2017.06)	This method increases the exploration ability of the model by adding noise to the parameters.	Fortunato et al. [42]

**Table 2:** MDP model parameter settings.

Markov Decision Model	Parameter Settings
Number of signal sub-band divisions	M=5
Number of sub-band energy divisions	N=5
State space	$S=(s_{1},s_{2},s_{3},\cdots ,s_{6^{5}})$
Action space	$A=(a_{1},a_{2},a_{3},\cdots ,a_{6^{5}})$
Single state	$s_{t}=[\alpha_{1},\alpha_{2},\alpha_{3},\alpha_{4},\alpha_{5}],\alpha \in [0,5]$
Single action	$a_{t}=[\beta_{1},\beta_{2},\beta_{3},\beta_{4},\beta_{5}],\beta \in [0,5]$
γ	0.9

**Table 3:** Simulation parameters.

A Certain Foreign Radar	Simulation Parameter Settings
Working band	X-band
Centre frequency	9.5 GHz
Signal bandwidth	100 MHz
Sub-band bandwidth	20 MHz
Target aircraft speed	250 m/s
$\sigma_{h}^{2}(f)$	$\left\{25,\;4,\;16,\;64,\;49\right\}$
Clutter	$\left\{0.8,\;0.2,\;1.0,\;3.0,\;4.5\right\}$
Noise	1

## Data Availability

Not applicable.

## Abbreviations

The following abbreviations are used in this manuscript:

DQN	deep Q network
DRL	deep reinforcement learning
D3QN	duelling double deep Q network
IFFT	inverse fast Fourier transform
ITM	iterative transform method
LFM	linear frequency modulation
MDP	Markov decision process
RL	reinforcement learning
SJNR	signal-to-jamming plus noise ratio
TD error	time difference error

## References

[1] Li Z., Tang B., Zhou Q., Shi J., Zhang J. (2022). A review of research on the optimal design of new system airborne radar waveforms. Syst. Eng. Electron. Technol..

[2] Zhao G. (2015). Principles of Radar Countermeasures.

[3] Skolnik M. (2010). Translated by Nanjing Electronics Technology Research Institute. Radar Handbook.

[4] Chen X., Xue Y., Zhang L., Huang Y. (2021). Airborne Radar System and Information Processing.

[5] Haykin S. Cognitive radar networks. Proceedings of the 2005 1st IEEE International Workshop on Computational Advances in Multi-Sensor Adaptive Processing.

[6] Yan Y., Chen H., Su J. Overview of anti-jamming technology in the main lobe of radar. Proceedings of the 2021 IEEE 4th International Conference on Automation, Electronics and Electrical Engineering (AUTEEE).

[7] Fang W., Quan Y., Sha M., Liu Z., Gao X., Xing M. (2021). Dense False Targets Jamming Suppression Algorithm Based on the Frequency Agility and Waveform Entropy. Syst. Eng. Electron..

[8] Quan Y., Fang W., Sha M., Chen M., Ruan F., Li X., Meng F., Wu Y., Xing M. (2021). Status and Prospects of Frequency Agile Radar Waveform Countermeasure Technology. Syst. Eng. Electron..

[9] Yan F., Su J., Li Y. (2021). Research and Test of Radar Low Intercept Waveform Anti-Main Lobe Jamming Technology. Fire Control. Radar Technol..

[10] Wei S., Zhang L., Liu H. (2022). Joint frequency and PRF agility waveform optimization for high-resolution ISAR imaging. IEEE Trans. Geosci. Remote Sens..

[11] Ou J., Li J., Zhang J., Zhan R. (2020). Frequency Agility Radar Signal Processing.

[12] Thornton C., Buehrer R.M., Martone A. (2021). Constrained contextual andit learning for adaptive radar waveform selection. IEEE Trans. Aerosp. Electron. Syst..

[13] Cui G., De M., Farina A., Li J. (2020). Radar Waveform Design Based on Optimization Theory.

[14] Xia D., Zhang K., Ding B., Li B. (2022). Anti-simultaneous jamming based on phase-coded waveform shortcuts and CFAR techniques. Syst. Eng. Electron..

[15] Hu H., Lui R., Zhang J. An improved radar anti-jamming method. Proceedings of the 2018 IEEE 3rd International Conference on Signal and Image Processing.

[16] Wang H., Yang X., Li Y. Radar anti-retransmitted jamming technology based on agility waveforms. Proceedings of the 2019 IEEE International Conference on Signal, Information and Data Processing (ICSIDP).

[17] Zhou C., Liu F., Liu Q. (2017). An Adaptive Transmitting Scheme for Interrupted Sampling Repeater Jamming Suppression. Sensors.

[18] Gong X., Meng H., Wei Y., Wang X. (2011). Phase-Modulated Waveform Design for Extended Target Detection in the Presence of Clutter. Sensors.

[19] Zhang Y., Wei Y. (2018). A cognitive-based approach to the design of anti-folding extended clutter waveforms. Syst. Eng. Electron..

[20] He J., Cheng Z., He Z. (2021). Cognitive constant-mode waveform design method for resisting intermittent sampling and forwarding jamming. Syst. Eng. Electron..

[21] Hao T., Hu S., Gao W., Li J., Cao X., Wang P. (2022). Detection-oriented low peak-to-average ratio robust waveform design for cognitive radar. Electron. Inf. Warf. Technol..

[22] Yu R., Yang W., Fu Y., Zhang W. (2022). A Review of Cognitive Waveform Optimisation for Different Radar Tasks. Acta Electron. Sin..

[23] Wei Y., Meng H., Liu Y., Wang X. (2010). Extended Target Recognition in Cognitive Radar Networks. Sensors.

[24] Ailiya, Wei Y., Yuan Y. Reinforcement learning-based joint adaptive frequency hopping and pulse-width allocation for radar anti-jamming. Proceedings of the 2020 IEEE Radar Conference.

[25] Liu K., Lu X., Xiao L., Xu L. Learning based energy efficient radar power control against deceptive jamming. Proceedings of the 2020 IEEE Global Communications Conference.

[26] Wang H., Wang F. (2020). Application of a reinforcement learning algorithm in intelligent anti-jamming of radar. Mod. Radar.

[27] Ahmed A., Ahmed A., Fortunati S., Sezgin A., Greco M., Gini F. (2021). A reinforcement learning based approach for multitarget detection in massive MIMO radar. IEEE Trans. Aerosp. Electron. Syst..

[28] Li K., Jiu B., Liu H., Liang S. Reinforcement Learning based Anti-jamming Frequency Hopping Strategies Design for Cognitive Radar. Proceedings of the 2018 IEEE International Conference on Signal Processing, Communications and Computing (ICSPCC).

[29] Li K., Jiu B., Liu H. Deep Q-network based anti-jamming strategy design for frequency agile radar. Proceedings of the 2019 International Radar Conference.

[30] Li K., Jiu B., Wang P., Liu H., Shi Y. (2021). Radar Active Antagonism through Deep Reinforcement Learning: A Way to Address The Challenge of Mainlobe Jamming. Signal Process..

[31] Ak S., Brüggenwirth S. Avoiding Jammers: A Reinforcement Learning Approach. Proceedings of the 2020 IEEE International Radar Conferenc (RADAR).

[32] Selvi E., Buehrer R., Martone A., Sherbondy K. On The Use of Markov Decision Processes in Cognitive Radar: An Application to Target Tracking. Proceedings of the 2018 IEEE Radar Conference (RadarConf18).

[33] Kozy M., Yu J., Buehrer R., Martone A., Sherbondy K. Applying Deep-Q Networks to Target Tracking to Improve Cognitive Radar. Proceedings of the 2019 IEEE Radar Conference (RadarConf).

[34] Shi Y., Jiu B., Yan J., Liu H., Li K. (2020). Data-Driven Simultaneous Multibeam Power Allocation: When Multiple Targets Tracking Meets Deep Reinforcement Learning. IEEE Syst. J..

[35] Hessel M., Modayil J., Hasselt H., Schaul T., Ostrovski G., Dabney W., Horgan D., Piot B., Azar M., Silver D. Rainbow: Combining improvements in deep reinforcement learning. Proceedings of the 32rd AAAI Conference on Artificial Intelligence.

[36] Watkins C., Dayan P. (1992). Q-learning. Mach. Learn.

[37] Mnih V., Kavukcuoglu K., Silver D., Graves A., Antonoglou I., Wierstra D., Riedmiller M. (2013). Playing Atari with Deep Reinforcement Learning. arXiv.

[38] Bellemare M.G., Dabney W., Munos R. A Distributional Perspective on Reinforcement Learning. Proceedings of the 34rd International Conference on Machine Learning (ICML 2017).

[39] Hasselt V.H., Guez A., Silver D. Deep Reinforcement Learning with Double Q-Learning. The 30rd AAAI Conference on Artificial Intelligence.

[40] Schaul T., Quan J., Antonoglou I., Silver D. (2015). Prioritized Experience Replay. arXiv.

[41] Wang Z., Schaul T., Hessel M., Hasselt H., Lanctot M., Freitas N. Dueling Network Architectures for Deep Reinforcement Learning. Proceedings of the 33rd International Conference on Machine Learning (ICML 2016).

[42] Fortunato M., Azar M.G., Piot B., Menick J., Osband I., Graves A., Mnih V., Munos R., Hassabis D., Pietquin O. (2017). Noisy Networks for Exploration. arXiv.

[43] Zheng Z., Li W., Zou K., Li Y. (2022). Anti-jamming Waveform Design of Ground-to-air Radar Based on Reinforcement Learning. Acta Armamentarii.

[44] Wang H., Li W., Wang H., Xu J., Zhao J. (2019). Radar Waveform Strategy Based on Game Theory. Radio Eng..

[45] Romero R., Bae J., Goodman N. (2011). Theory and Application of SNR and Mutual Information Matched Illumination Waveforms. IEEE Trans. Aerosp. Electron. Syst..

[46] Gagniuc P. (2017). Markov Chains: From Theory to Implementation and Experimentation.

[47] Steven M.K. (2014). Fundamentals of Statistical Signal Processing: Estimation and Detection Theory.

[48] Li X., Fan M. (2012). Research Advance on Cognitive Radar and Its Key Technology. Acta Electron. Sin..

[49] Jackson L., Kay S., Vankayalapati N. (2010). Iterative Method for Nonlinear FM Synthesis of Radar Signals. IEEE Trans. Aerosp. Electron. Syst..

